# Thymic Carcinoma Developing Years after Thymectomy

**Published:** 2017

**Authors:** Ali Ghorbani-Abdehgah, Pegah Mohaghegh, Amir Hossein Latif, Farzad Fatehi, Ahmadreza Soroush

**Affiliations:** 1 Department of Surgery, Research Center for Improvement of Surgical Outcomes and Procedures, Shariati Hospital, Tehran University of Medical Sciences, Tehran, Iran,; 2 Research and Development Center of Shariati Hospital, Tehran University of Medical Sciences, Tehran, Iran,; 3 Iranian Center of Neurological Research.

Thymoma is the most common primary neoplasm of the anterior mediastinum (1). Most patients with thymoma are between the ages of 40 and 60. The prevalence of thymoma is similar in men and women (2). Between 30% to 50% of patients with thymoma suffer from Myasthenia Gravis (MG); whereas 10% to 15% of MG patients have thymoma (1). MG is an autoimmune disease and its symptoms include ptosis, extraocular muscle weakness, dysphagia, and limb weakness. The main symptom of MG is fluctuating weakness in any or all of the ocular, bulbar, limb, and respiratory muscles (3). In patients who have MG with a tumor in the mediastinum, thymectomy is widely used. During thymectomy, the thymus gland, the tumor if it exists as well as the tissue surrounding it, and the tissue between the right and left phrenic nerves are removed (4). Herein, we present a patient with MG who was found to have invasive thymic carcinoma years after thymectomy for apparently thymoma .

The patient in this study was a 60-year-old man diagnosed with MG in 1984. In November 2016, he was referred to the neurology ward of Shariati Hospital with symptoms including nasal speech, bilateral ptosis, dyspnea, weakness in the face and neck muscles, and dysphagia. He had been hospitalized for similar symptoms 32 years ago, undergoing a thymectomy with median sternotomy incision. Following the operation, the patient’s drug regimen was gradually stopped. Since then, the patient had not required medication.

**Figure 1:**
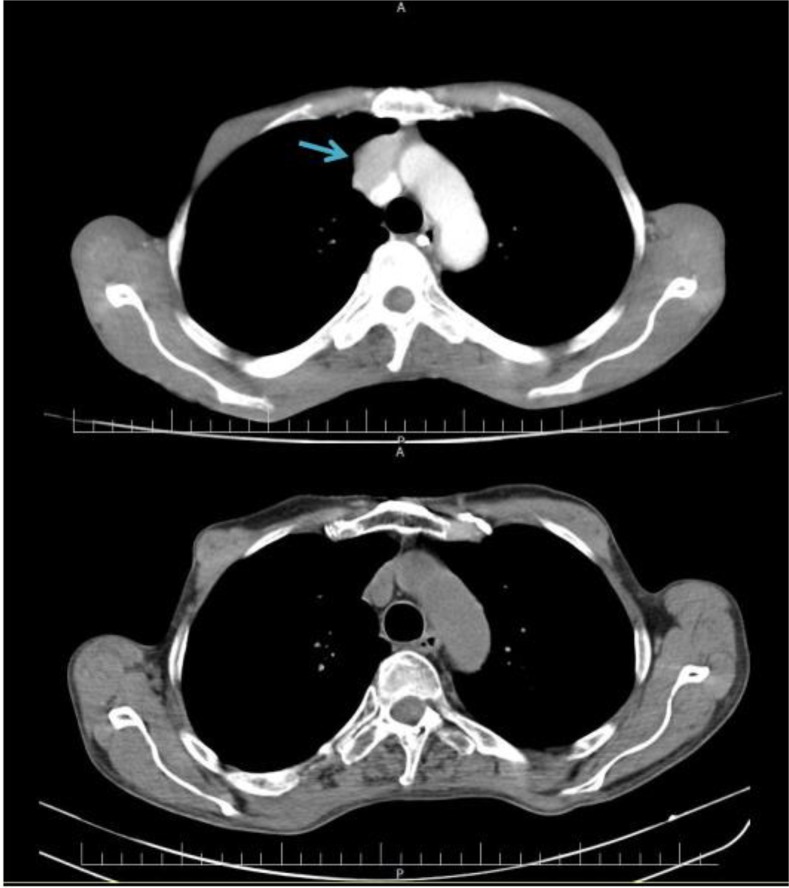
Preoperative (up) and post operative (down) spiral chest CT scan with contrast of a 60-year-old man with thymoma

Ten days before being referred to Shariati Hospital, the symptoms of MG reappeared. In this manifestation, they included nasal speech, diplopia, dysphagia, ptosis, a reduction in gag reflex, and weakness in left and right facial muscles. Motor strength was decreased in the proximal muscles of the lower limbs (4/5). Deep Tendon Reflexes (DTR) were 2+ (a brisk response; normal). Cerebellum sensory was normal. Anti-Acetylcholine antibody and EME-RNS (3- H2 repetitive nerve stimulation) tests returned positive results. In spiral CT-scan of the chest with contrast IV, a lobulated solid mass 34*28mm in size was discovered in the anterior mediastinum in the right side of the aortic arch and ascending aorta (Figure 1).

The patient’s drug regimen consisted of mestinon 60 mg QID, and prednisolone 50 mg daily. After the symptoms of MG were sufficiently controlled, the patient was referred for repeat surgery. Excision of mediastinal mass and other fatty and Lymphatic tissue around the mass was performed with posterolateral thoracotomy incision and from the fourth intercostal space.

Pathologic evaluation of the sample revealed the mass to be a well-differentiated thymic carcinoma. The patient was referred for adjuvant treatment, subsequently undergoing 31 sessions of radiotherapy. One week after the operation, the patient returned with complains of severe diarrhea, which was solved through a reduction in the mestinon dosage. Thereafter, general condition of the patient improved and medications were gradually tapered.

Thymic tumors account for 20% of anterior mediastinal neoplasms in adults and Thymic carcinoma accounts for 5% of thymic malignancies. It remains unclear whetherthymectomy reduces the risk of developing a malignancy. Thymic tumors can recur after thymectomy. Transformation to malignant thymic carcinoma has been reported but is rare (5). About 1% of thymic carcinoma patients exhibit MG symptoms (6). In this case, resurgence of MG was accompanied with the reappearance of thymic carcinoma and in similar cases care must be taken to search for the possibility of a tumor.Our findings fortify the points that complete resection of thymus, tumor, and other tissues between right and left phrenic nerves is very important. Thus, in patients with a deterioration in MG status after several years of disease stability, a chest CT is required to evaluate for development of thymic carcinoma or thymoma.
